# Digoxin Downregulates NDRG1 and VEGF through the Inhibition of HIF-1α under Hypoxic Conditions in Human Lung Adenocarcinoma A549 Cells

**DOI:** 10.3390/ijms14047273

**Published:** 2013-04-02

**Authors:** Dong Wei, Jing-Jing Peng, Hui Gao, Hua Li, Dong Li, Yong Tan, Tao Zhang

**Affiliations:** Oncology Medicine Centre, General Hospital of Chinese PLA Chengdu Command, Chengdu 6l0083, China; E-Mails: weidongchengdu@163.com (D.W.); jingjingpeng2012@163.com (J.-J.P.); gaohuichengdu@163.com (H.G.); lihuachengdu@163.com (H.L.); lidongchengdu@163.com (D.L.); yongtan2012@126.com (Y.T.)

**Keywords:** digoxin (DGX), A549 cells, hypoxia, VEGF, NDRG1, HIF-1α

## Abstract

Digoxin, an inhibitor of Na^+^/K^+^ ATPase, has been used in the treatment of heart-related diseases (such as congestive heart failure and atrial arrhythmia) for decades. Recently, it was reported that digoxin is also an effective HIF-1α inhibitor. We investigated whether digoxin could suppress tumor cell growth through HIF-1α in non-small cell lung cancer cells (A549 cells) under hypoxic conditions. An MTT assay was used to measure cell viability. RT-PCR and western blotting were performed to analyze the mRNA and protein expression of VEGF, NDRG1, and HIF-1α. HIF-1α nuclear translocation was then determined by EMSA. Digoxin was found to inhibit the proliferation of A549 cells under hypoxic conditions. Our results showed that hypoxia led to the upregulation of VEGF, NDRG1, and HIF-1α both at the mRNA and protein levels. We also found that the hypoxia-induced overexpression of VEGF, NDRG1, and HIF-1α was suppressed by digoxin in a concentration-dependent manner. As expected, our EMSA results demonstrated that under hypoxic conditions HIF-1α nuclear translocation was also markedly reduced by digoxin in a concentration-dependent manner. Our results suggest that digoxin downregulated hypoxia-induced overexpression of VEGF and NDRG1 at the transcriptional level probably through the inhibition of HIF-1α synthesis in A549 cells.

## 1. Introduction

Lung cancer is the most commonly diagnosed forms of cancer and is the leading cause of cancer mortality worldwide, with non-small cell lung cancer (NSCLC) constituting approximately 80% of primary bronchial lung cancers [1,2].

The cellular concentration of oxygen plays a critical role in regulating the expression of 50–500 or more genes involved in cell proliferation, angiogenesis, modulation, glucose metabolism, survival, and invasion in solid tumors during tumor progression and metastasis [3,4]. Hypoxia leads to the reduced effectiveness of chemotherapy and radiation treatment in tumors [5,6]. Under hypoxic conditions, transcription factor hypoxia-inducible factor-1 (HIF-1) is considered a critical regulator of the adaptation responses of tumor cells [7–9]. HIF-1, a heterodimeric transcription factor, consists of 2 subunits, HIF-1α and HIF-1β. Of these, HIF-1α is regulated by oxygen-dependent prolyl hydroxylation in the ODD region [7–10]. Normally, under normoxia conditions, HIF-1α is continually degraded though ubiquitinylation by E3 ubiquitin-protein ligases. However, this degradation mechanism is suppressed by hypoxia, resulting in the accumulation of HIF-1α in cell and translocation into the nucleus to bind to HIF-1β, thus forming the HIF-1α/HIF-1β heterodimer, HIF-1. HIF-1 then binds to the hypoxia-response element (HRE), which is the enhancer that upregulates target gene expression [7–10].

Vascular endothelial growth factor (VEGF) is a critical growth factor in the regulation of angiogenesis. Several studies have shown that VEGF is upregulated in tumors, including in NSCLCs [11,12]. Angiogenesis is an indispensible process in tumor growth and metastasis [13]. According to previous reports, HRE has been identified on the VEGF gene [14,15]. It has also been shown that HIF-1 is an important regulator of VEGF under hypoxic conditions in A549 cells [16]. Similarly, studies have confirmed that HRE is also found on the N-Myc downregulated gene 1 (NDRG1) [17,18]. Moreover, it has also been shown that the HIF-1 signaling pathway contributed substantially to the regulation of NDRG1 under hypoxic conditions [17,18].

Recently, a study demonstrated that cardiac glycosides (such as digoxin and ouabain) are powerful inhibitors of HIF-1α in tumors [19]. Taken together, we hypothesized that the hypoxia-induced overexpression of VEGF and NDRG1 would be downregulated by digoxin through the inhibition of HIF-1α in A549 cells.

## 2. Results and Discussion

### 2.1. Digoxin Inhibits the Viability of A549 Cells

**Figure 1:**
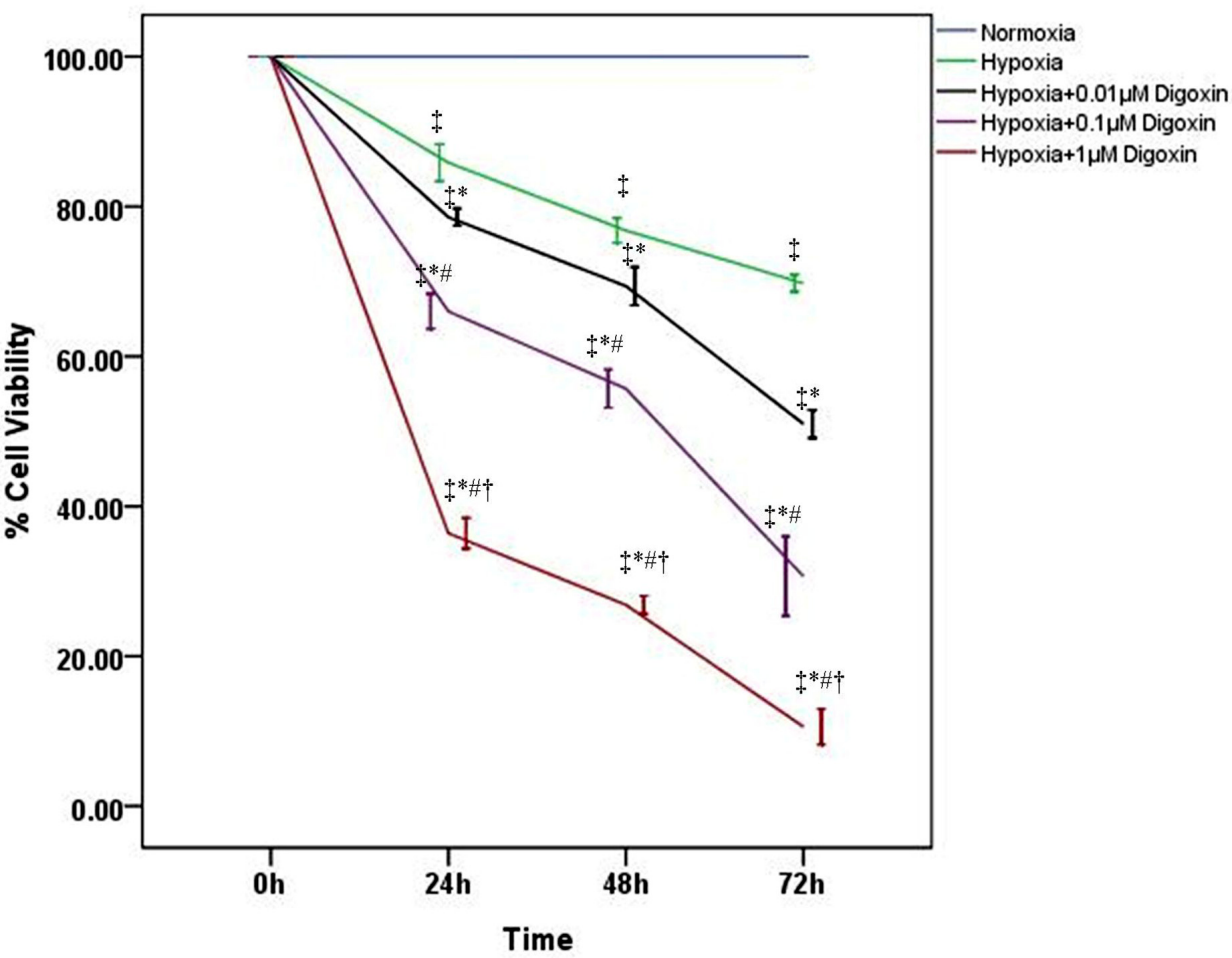
Digoxin inhibits the viability of A549 cells under hypoxic conditions. A549 cell viability was measured by MTT assay and analyzed at different time points (0, 24, 48 and 72 h) with or without various concentrations of digoxin (0.01, 0.1, and 1 μM). Quantitative data are presented as mean ± SD (*n* = 4). * *p* < 0.01 compared with hypoxia at the corresponding time points. # *p* < 0.01 compared with hypoxia + 0.01 μM digoxin at the corresponding time points. † *p* < 0.01 compared with hypoxia + 0.1 μM digoxin at the corresponding time points. ‡ *p* < 0.01 compared with normoxia at the corresponding time points.

To determine the effect of digoxin on A549 cell proliferation in hypoxia, cells were treated with or without various concentrations of digoxin. Our MTT results showed that under hypoxic conditions, the viability of A549 cells was reduced by digoxin in a time- and concentration-dependent manner (Figure 1), whereas the cell viabilities under normoxic conditions were markedly higher than those under hypoxia (Figure 1).

### 2.2. Digoxin Attenuates mRNA and Protein Expression of VEGF and NDRG1 in A549 Cells

**Figure 2:**
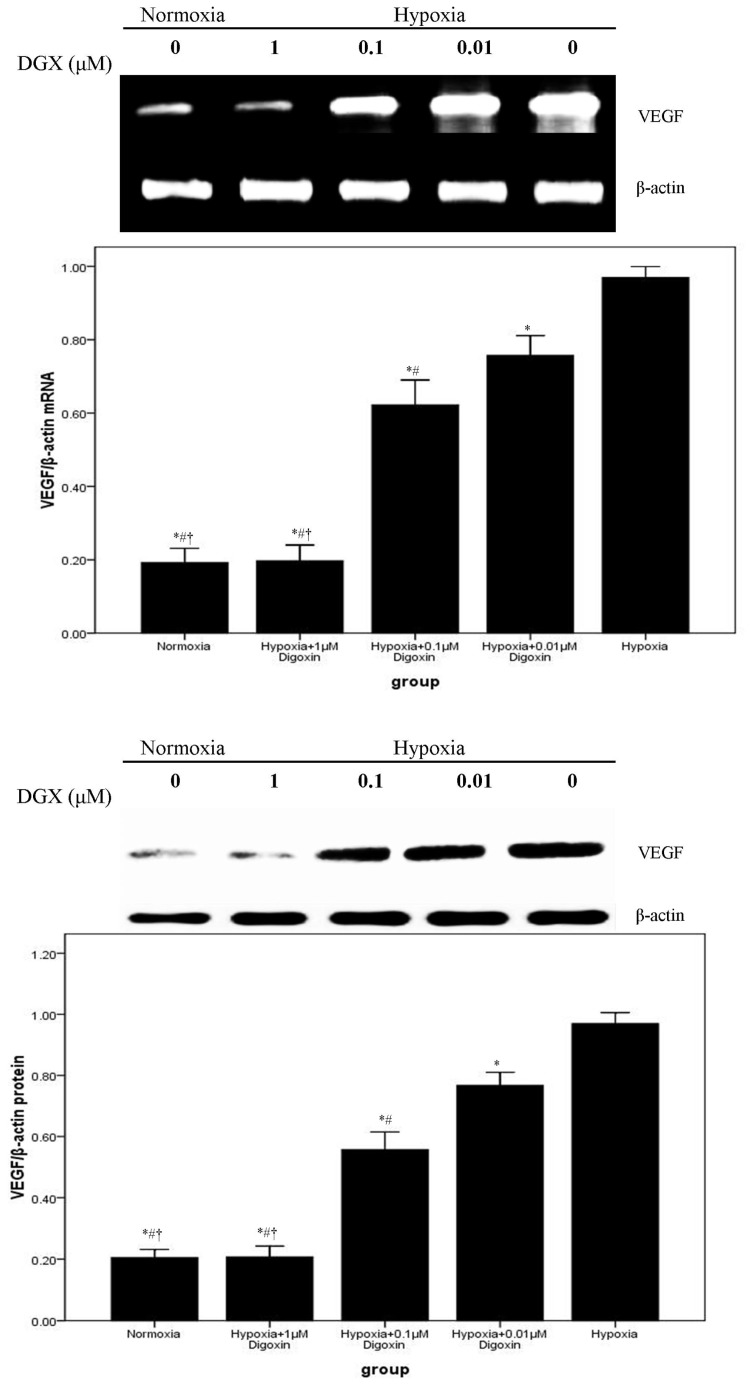
Digoxin attenuates mRNA and protein expression of VEGF in A549 cells under hypoxic conditions. Under normoxic or hypoxic conditions, mRNA and protein levels of VEGF in A549 cells treated with or without various concentrations of digoxin (0.01, 0.1, and 1 μM) for 24 h were analyzed. Quantitative data were presented as mean ± SD (*n* = 4). * *p* < 0.05 compared with hypoxia. # *p* < 0.05 compared with hypoxia + 0.01 μM digoxin. † *p* < 0.05 compared with hypoxia + 0.1 μM digoxin.

**Figure 3:**
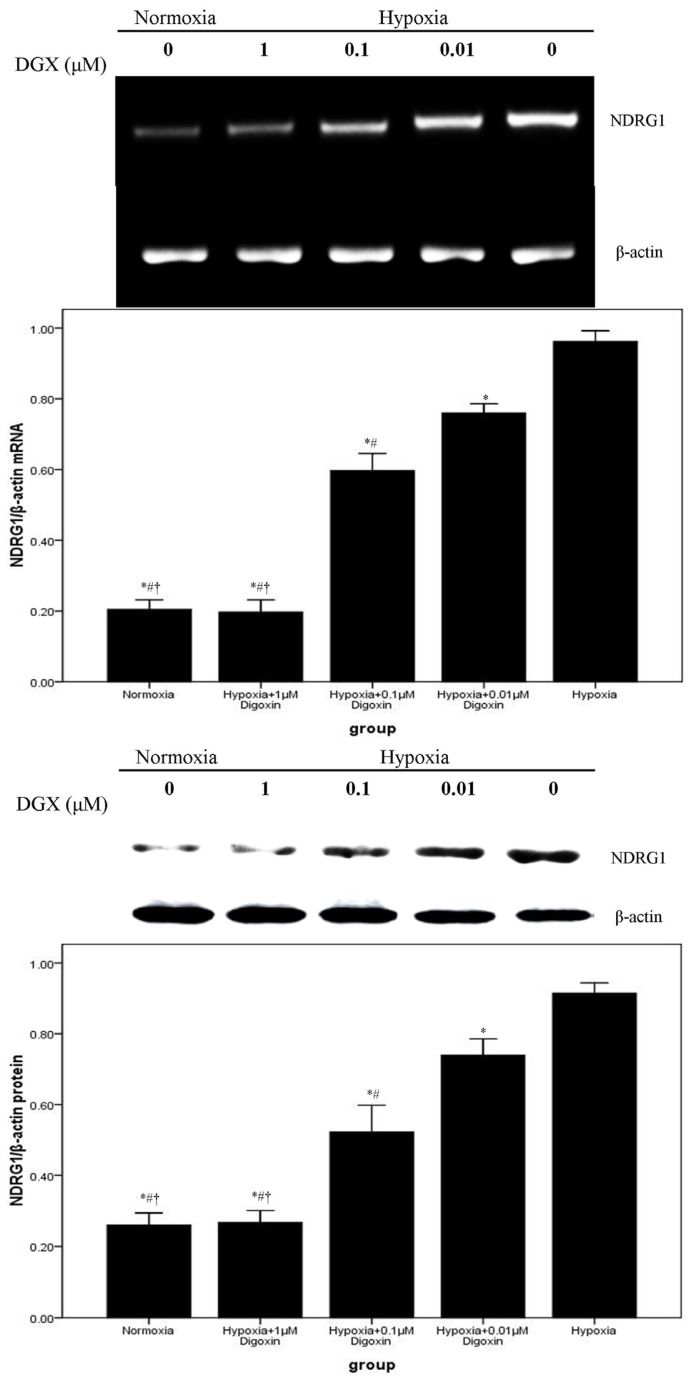
Digoxin inhibits mRNA and protein expression of NDRG1 in A549 cells under hypoxic conditions. Under normoxic or hypoxic conditions, mRNA and protein levels of NDRG1 in A549 cells treated with or without various concentrations of digoxin (0.01, 0.1, and 1 μM) for 24 h were measured. Quantitative data were presented as mean ± SD (*n* = 4). * *p* < 0.05 compared with hypoxia. # *p* < 0.05 compared with hypoxia + 0.01 μM digoxin. † *p* < 0.05 compared with hypoxia + 0.1 μM digoxin.

mRNA and protein expression levels of VEGF and NDRG1 were evaluated by RT-PCR and western blotting. Our data demonstrated that VEGF and NDRG1 were significantly upregulated in A549 cells after exposure to hypoxic conditions. However, the hypoxia-induced overexpression of VEGF and NDRG1 was inhibited by digoxin in a concentration-dependent manner. Furthermore, the expression of VEGF and NDRG1 in A549 cells treated with 1 μM digoxin showed no difference from cells under normoxia (Figures 2 and 3).

### 2.3. Digoxin Suppresses mRNA and Protein Expression of HIF-1α and Reduces the Level of HIF-1/DNA Complex in A549 Cells

**Figure 4:**
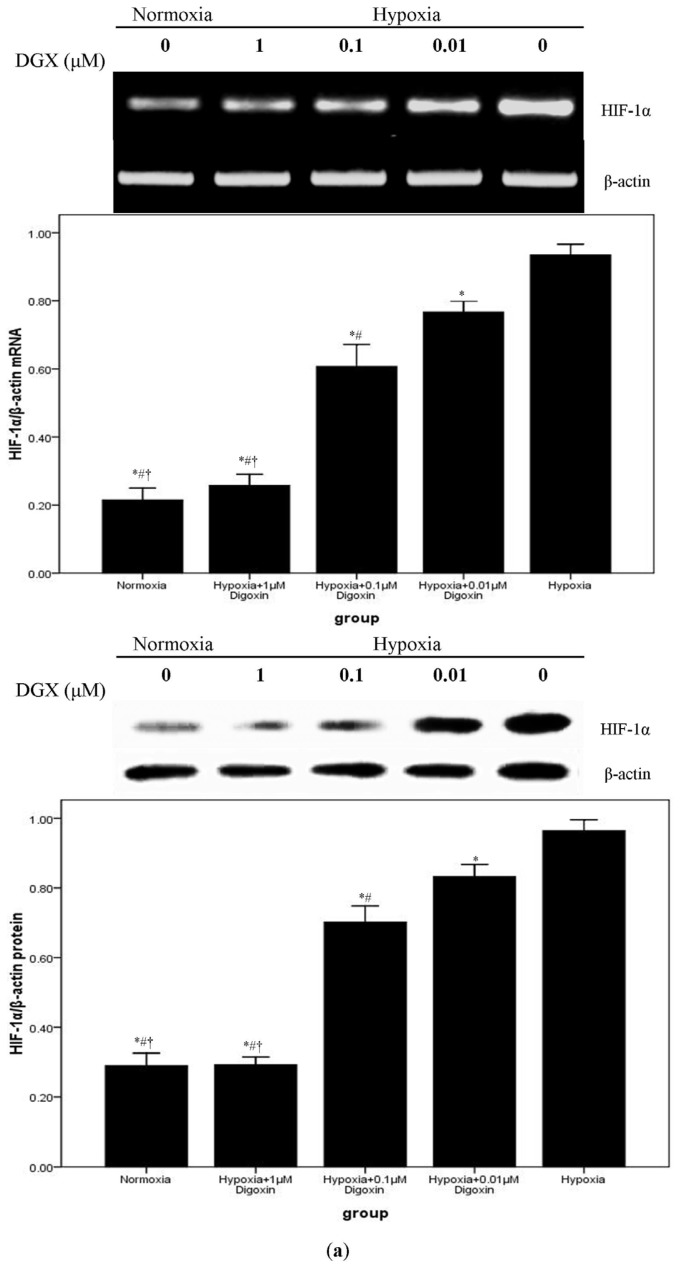

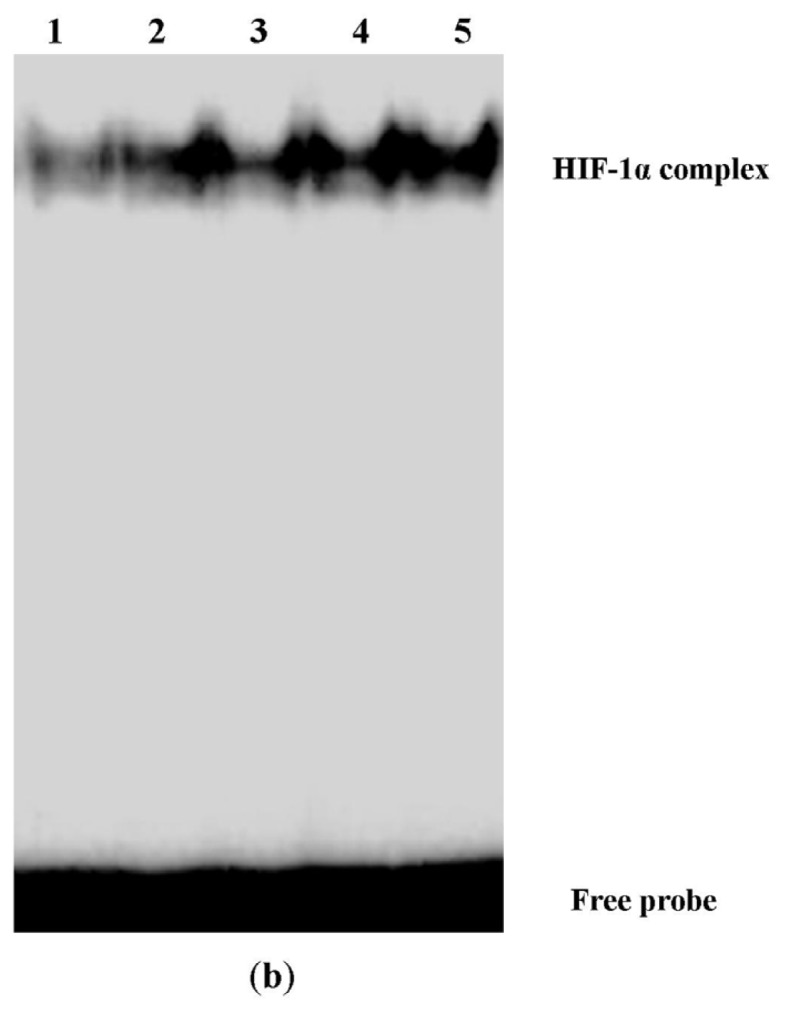
Digoxin suppresses mRNA and protein expressions of HIF-1α and reduces the HIF-1/DNA complex in A549 cells under hypoxic conditions. (**a**) Under normoxic or hypoxic conditions, mRNA and protein levels of HIF-1α in A549 cells treated with or without various concentrations of digoxin (0.01, 0.1, and 1 μM) for 24 h were determined. Quantitative data were presented as mean ± SD (*n* = 4). * *p* < 0.05 compared with hypoxia. # *p* < 0.05 compared with hypoxia + 0.01 μM Digoxin. † *p* < 0.05 compared with hypoxia + 0.1 μM Digoxin. (**b**) EMSA was used to detect HIF-1α nuclear translocation. Results shown are from 4 repeated, individual experiments. 1: Normoxia; 2: Hypoxia + 1 μM Digoxin; 3: Hypoxia + 0.1 μM Digoxin; 4: Hypoxia + 0.01 μM Digoxin; 5: Hypoxia.

We performed RT-PCR and western blotting to determine mRNA and protein expressions of HIF-1α. Our findings indicated that HIF-1α production was markedly increased by hypoxia stimulation in A549 cells. However, hypoxia-enhanced HIF-1α production was inhibited by digoxin in a concentration-dependent manner. Moreover, as shown in Figure 4a, no difference in HIF-1α production was noted between cells subjected to normoxia and those subjected to hypoxia + 1 μM digoxin. A DNA probe derived from the promoter of the *VEGF* gene, including a HIF-1α binding site, was used for EMSA to discover the binding activity of HIF-1α in the nuclear extracts prepared from cells treated with or without digoxin. Our result showed that the HIF-1/DNA complex was strongly increased after exposure to hypoxia. However, under hypoxic conditions, the HIF-1/DNA complex was reduced by digoxin in a concentration-dependent manner (Figure 4b).

The purpose of the present study was to explore whether digoxin would inhibit the synthesis of HIF-1α and its regulated genes, *VEGF* and *NDRG1*, in A549 cells under hypoxic conditions.

NSCLC is the most prevalent type of lung cancer. Despite advances in surgery, chemotherapy, and radiotherapy, the overall survival rate of patients with NSCLC is still poor [1,2].

HIF-1 is a critical transcription factor involved in tumor cell proliferation and adaptation in hypoxia. HIF-1 is a heterodimer consisting of the oxygen-sensitive subunit HIF-1α and the constitutively expressed subunit HIF-1β [3,4,6]. The expression of HIF-1α is regulated by oxygen concentration. Under normoxic conditions, HIF-1α is continually degraded by ubiquitination and proteosomal degradation. HIF-1α is hydroxylated by the oxygen-dependent enzyme prolyl hydroxylase enzyme (PHD). Thus, under hypoxic conditions, HIF-1α binding to the von Hippel-Lindau protein (VHL) constitutes a part of the E3-ubiqutin lipase complex and abolishes degradation. Therefore, hypoxia promotes the stability and accumulation of HIF-1α, resulting in the translocation of HIF-1α to the nucleus and interaction with HIF-1β. Once the HIF-1α/HIF-1β heterodimer (HIF-1) is formed, it binds to the enhancer region HRE in hypoxia-sensitive target genes, leading to activation [7–11].

Previous reports describe that overexpression of HIF-1α is a common event in multiple types of carcinomas, which has also been associated with aggressive tumor behavior, therapeutic resistance, and overall poor prognosis [8,20]. HIF-1α has been considered as a potential therapeutic target of several cancers [8,20]. A number of genes involved in cell proliferation, cell survival, angiogenesis, tumor cell immortalization, stem cell maintenance/de-differentiation, genetic instability, autocrine growth, invasion/metastasis, and treatment failure, are upregulated under hypoxic conditions through the activation of HIF-1 [7,8,21].

Digoxin, one of the cardiac glycosides, is an inhibitor of Na^+^/K^+^ ATPase that has been used for a few decades to treat patients with congestive heart failure and arrhythmias [20,22]. Increasing evidence has shown significant anticancer effects mediated by cardiac glycosides [23]. A series of epidemiological studies by Stenkvist *et al.* compared breast cancer tissues from women treated with digitalis for cardiac diseases to tissue samples from control patients; their results demonstrated that women on digitalis therapy developed more benign forms of breast tumors than did control patients [23]. Recently, Zhang, H *et al.* indicated that anti-tumor effects of cardiac glycosides result from the inhibition of the synthesis of HIF-1α in tumor under hypoxic condition [19]. Simultaneously, many studies have proved that HIF-1 plays a critical role in A549 cells proliferation under hypoxic conditions [16,24]. In our investigation, the viabilities of A549 cells were determined by MTT assay. Our data showed that under hypoxic conditions, digoxin reduced the viability of A549 cells in a concentration- and time-dependent manner (Figure 1), indicating the potent effect of digoxin on the suppression of tumor cell growth. Furthermore, our results, which are consistent with those of the previous study, showed that hypoxia slightly reduced A549 cell viability compared with the cells under normoxic conditions (Figure 1) [21].

Solid tumor growth requires the formation of new blood vessels. Angiogenesis is an indispensible process in tumor growth and metastasis [4,5]. In the tumor mass, dense vascular networks often lead to the growth of human tumors [4,5]. The study found that the tumor mass does not keep expanding without angiogenesis, which would otherwise exhaust the available oxygen supply leading to hypoxia [5]. In 1971, Folkman, known as the “father of tumor angiogenesis,” proposed that inhibiting angiogenesis might be an effective anti-tumor strategy [25]. VEGF is a critical growth factor in the regulation of angiogenesis. VEGF was first discovered by Senger and colleagues as a vascular permeability factor secreted by a guinea pig tumor cell line [26]. A number of studies showed that VEGF was upregulated in tumors, including NSCLC [12,14,16]. According to the reports, HRE was identified on the *VEGF* gene. It has also been shown that HIF-1 is an important regulator of VEGF in A549 cells under hypoxic conditions [14,15]. In our investigation, we found that VEGF production in A549 cells was markedly upregulated after exposure to hypoxic conditions (Figure 2). Our results also demonstrated that digoxin suppressed the hypoxia-induced overexpression of VEGF in A549 cells in a concentration-dependent manner (Figure 2).

NDRG1, also known as Cap43, Drg 1, RTP, and rit42 following the discovery of its gene (NDRG1) in different laboratories, is a stress responsive protein that shuttles between the cytoplasm and nucleus upon certain conditions [27]. Similarly, 3 HIF-1 binding sites have been identified on the non-coding sequence of the *NDRG-1* gene, one in its promoter and the other 2 in the 3′ untranslated region [28]. Moreover, the results of the present study also indicate that the HIF-1 signaling pathway contributes substantially to the regulation of NDRG1 under hypoxic conditions [29]. NDRG1 has been shown to possess many specific characteristics for clinical analysis and identification purposes. There has been more interest in NDRG1 as a marker of tumor progression and as an enhancer of cellular differentiation [29]. Accordingly, our data suggested that NDRG1 expression was markedly increased under hypoxic conditions in A549 cells. We also found that digoxin inhibited this hypoxia-induced upregulation of NDRG1 in a concentration-dependent manner (Figure 3). Experiments conducted in this study provide clear evidence that hypoxia promoted the expression of VEGF and NDRG1, both at the mRNA and protein levels. These findings also suggest that the tumor cell inhibitory effect of digoxin truly results from the downregulation of VEGF and NDRG1.

According to a previous report, the anti-tumor effect of digoxin possibly resulted from the inhibition of the synthesis of HIF-1α at the protein level, without suppressing the mRNA expression of HIF-1α in Hep 3B and PC 3 cells [19]. Therefore, we analyzed the production of HIF-1α. First, we found that hypoxia notably upregulated HIF-1α production in A549 cells. Subsequently, our data also showed that hypoxia-induced overexpression of HIF-1α was reduced by digoxin in a concentration-dependent manner (Figure 4a). However, the findings showed that both the mRNA and protein levels of HIF-1α were significantly reduced at the same time. We assumed that the different results from the 2 studies were due to different cell lines and their different biological characters [30,31].

In addition, the EMSA result also demonstrated that the hypoxia-enhanced nuclear translocation of HIF-1 was inhibited by digoxin in a concentration-dependent fashion (Figure 4b). These data indicate that digoxin is a potent HIF-1α inhibitor through the reduced synthesis of HIF-1α in A549 cells. Nevertheless, the mechanisms underlying digoxin or other cardiac glycoside-associated signaling pathways in HIF-1α regulation remain unclear. Recently, Wang Y, *et al.* deduced that cardiac glycosides can induce autophagy in human NSCLC through the regulation of the mTOR and ERK1/2 signaling pathways [32]. Prassas I *et al.* demonstrated that digitoxin, another type of cardiac glycoside, induced cytotoxicity in cancer cells, which resulted from distinct kinase and interferon signaling networks [33]. Thus, further work should be carried out to understand the signaling pathways in HIF-1α regulation caused by cardiac glycosides under hypoxic conditions.

## 3. Experiment Section

### 3.1. Reagents

Human lung adenocarcinoma A549 cells were obtained from American Type Culture Collection (ATCC). Digoxin was obtained from Sigma Chemical Co (St. Louis, MO, USA). The MTT assay was purchased from Promega (Madison, WI, USA). Trizol reagent was purchased from Invitrogen (Grand Island, NY, USA). The first strand cDNA was synthesized using a reverse transcription kit (Shanghai Sangon, Shanghai, China). Anti-β-actin (mouse anti-human IgG), anti-HIF1α (mouse anti-human IgG), anti-VEGF (mouse anti-human IgG), and anti-NDRG1 (mouse anti-human IgG) antibodies were all purchased from Santa Cruz Biotechnology, Inc (Santa Cruz, CA, USA). The Lightshift kit from Pierce (Rockford, IL, USA) was used for EMSA.

### 3.2. Cell Culture and MTT Assay

A549 cells were cultured in RPMI 1640 supplemented with penicillin (100 IU/mL), streptomycin (100 mg/mL), and 10% heat-inactivated fetal bovine serum in a humidified atmosphere of 95% air and 5% CO_2_ at 37 °C. A549 cells were then plated at a density of 5 × 10^3^ cells per well in a 96-well plate. Hypoxia treatment was performed by placing cells in a CO_2_ Water Jacketed Incubator (Heracell 150i; Thermo Scientific, Waltham, MA, USA) flushed with a mixture of 1% O_2_, 5% CO_2_ and 94% N_2_. Cells were treated with different concentrations of digoxin (0.01, 0.1 and 1 μM) in 0.5% DMSO. Protein and mRNA were extracted from cells at specified time points (0, 24, 48 and 72 h), and cell proliferation was measured by MTT assay. Cell viability (%) = (OD sample/OD normoxia 0 h) × 100 (%).

### 3.3. RT-PCR

Total RNA was isolated from the A549 cells with Trizol reagent (Invitrogen, Grand Island, NY, USA). PCR was performed with a DNA thermal cycler in a 50-μL reaction volume, containing 5 μL 10× Taq Buffer, 4 μL of 2.5 mM dNTP, 4 μL of 25 mM MgCl_2_, 2 μL each forward and backward primers, 0.5 μL Taq polymerase, and 2 μL cDNA template, for 35 cycles by using a GeneAmp PCR system 9700 (Applied Biosystems, Foster City, CA, USA). The primer sequences were as follows: β-actin, (forward) 5′-CTCCATCCTGGCCTCGCTGT-3′ and (reverse) 5′-GCTGTCACCTTCACCGTTCC-3′; VEGF, (forward) 5′-TGCCCGCTGCTGTCTAAT-3′ and (reverse) 5′-TCTCCGCTCTGAGCAAGG-3′; NDRG1, (forward) 5′-AGGCAGGTGACAGCAGGGAC-3′ and (reverse) 5′-CGTGGCAGACGGCAAAGT-3′; HIF-1α, (forward) 5′-GCACAGGCCACATTCACG-3′ and (reverse) 5′-TGAAGATTCAACCGGTTTAAGGA-3′. The β-actin housekeeping gene was used as an internal control.

### 3.4. Western Blotting

Western blotting was used to measure the protein content. A549 cells were washed twice with cold PBS. Cells were then lysed with SDS sample buffer containing 50 mM Tris (pH 7.4), 2% SDS (*w*/*v*, 5% 2-mercaptoethanol and 10% glycerol. Cell homogenates were centrifuged at 10,000 rpm at 4 °C for 60 min. The protein concentrations for each sample were measured with a bicinchoninic acid assay kit by using BSA as the standard (Pierce, Rockford, IL, USA). Protein lysates (150 μg) from each sample were subjected to SDS-PAGE on a 10% acrylamide gel, and the separated proteins were transferred onto a PVDF membrane. After incubation for 1 h in blocking solution (5% dry milk in Tris-buffered saline with Tween 20) at room temperature, the membrane was incubated for 24 h with anti-β-actin (1:1000), anti-VEGF (1:500), anti-NDRG1 (1:500), or anti-HIF1α (1:500) at 4 °C. The secondary antibody (horseradish peroxidase-conjugated goat anti-mouse immunoglobulin) was added at a 1:10,000 dilution and incubated at room temperature for 1 h. Peroxidase labeling was detected with the enhanced chemoluminescence western blotting detection system (Amersham Pharmacia Biotech, NJ, USA) and analyzed by densitometry. The relative protein level was normalized to β-actin.

### 3.5. Electrophoretic Mobility Shift Assay (EMSA)

EMSA was performed according to the manufacturer’s instructions. Briefly, the oligonucleotide probe (5′-CCACAGTGCATACGTGGGCTCCAACA-3′ and 5′-TGTTGGAGCCCACGTATGCACT GTGG-3′), corresponding to the promoter in the human *VEGF* gene, was used. The oligonucleotides were annealed, and the resultant probes were end-labeled using a Biotin 3′ End DNA labeling Kit (Thermo Fisher Scientific, Rockford, IL, USA). Nuclei were isolated, and nuclear extracts were prepared from A549 cells using a Nuclear Extract Kit (Activemotif, Carlsbad, CA, USA). Binding reactions were performed using a LightShift Chemoluminescent EMSA Kit (Pierce Biotechnology, Rockford, IL, USA). The binding reactions containing nuclear protein (10 μg), Tris (10 mM), KCl (50 mM), DTT (1 mM), MgCl_2_ (5 mM), 2 μg poly (dI·dC) and 2 pmol of oligonucleotide probe were incubated for 20 min at room temperature. Specific binding was confirmed by a 200-fold excess of unlabeled probe used as a specific competitor. Protein DNA complexes were separated on a 6% non-denaturing acrylamide gel, transferred to positively charged nylon membranes, and cross-linked using a Stratagene crosslinker. Band shifts were visualized with a streptavidin-horseradish peroxidase followed by chemoluminescent detection.

### 3.6. Statistical Analysis

Statistical analyses were performed with SPSS version 17.0 software (IBM Corporation, Somers, NY, USA). All data in the present study were reported as mean ± SD. A Student’s *t*-test was used to measure significance, where *p* < 0.05 was considered to be statistically significant.

## 4. Conclusions

Taken together, our findings demonstrated that digoxin downregulated the hypoxia-induced overexpression of VEGF and NDRG1 in A549 cells at the transcriptional level, possibly through the inhibition of HIF-1α synthesis.
